# Structured reporting in radiology enables epidemiological analysis through data mining: urolithiasis as a use case

**DOI:** 10.1007/s00261-023-04006-9

**Published:** 2023-07-19

**Authors:** Tobias Jorg, Moritz C. Halfmann, Niklas Rölz, René Mager, Daniel Pinto dos Santos, Christoph Düber, Peter Mildenberger, Lukas Müller

**Affiliations:** 1 Department of Diagnostic and Interventional Radiology, University Medical Center of the Johannes Gutenberg-University Mainz, Langenbeckst. 1, 55131 Mainz, Germany; 2 Department of Urology, University Medical Center of the Johannes Gutenberg-University Mainz, Mainz, Germany; 3 Department of Radiology, University Hospital of Cologne, Cologne, Germany; 4 Department of Radiology, University Hospital of Frankfurt, Frankfurt, Germany

**Keywords:** Urolithiasis, Structured reporting, Epidemiology, Data mining

## Abstract

**Purpose:**

To investigate the epidemiology and distribution of disease characteristics of urolithiasis by data mining structured radiology reports.

**Methods:**

The content of structured radiology reports of 2028 urolithiasis CTs was extracted from the department’s structured reporting (SR) platform. The investigated cohort represented the full spectrum of a tertiary care center, including mostly symptomatic outpatients as well as inpatients. The prevalences of urolithiasis in general and of nephro- and ureterolithasis were calculated. The distributions of age, sex, calculus size, density and location, and the number of ureteral and renal calculi were calculated. For ureterolithiasis, the impact of calculus characteristics on the degree of possible obstructive uropathy was calculated.

**Results:**

The prevalence of urolithiasis in the investigated cohort was 72%. Of those patients, 25% had nephrolithiasis, 40% ureterolithiasis, and 35% combined nephro- and ureterolithiasis. The sex distribution was 2.3:1 (M:F). The median patient age was 50 years (IQR 36–62). The median number of calculi per patient was 1. The median size of calculi was 4 mm, and the median density was 734 HU. Of the patients who suffered from ureterolithiasis, 81% showed obstructive uropathy, with 2nd-degree uropathy being the most common. Calculus characteristics showed no impact on the degree of obstructive uropathy.

**Conclusion:**

SR-based data mining is a simple method by which to obtain epidemiologic data and distributions of disease characteristics, for the investigated cohort of urolithiasis patients. The added information can be useful for multiple purposes, such as clinical quality assurance, radiation protection, and scientific or economic investigations. To benefit from these, the consistent use of SR is mandatory. However, in clinical routine SR usage can be elaborate and requires radiologists to adapt.

**Graphical abstract:**

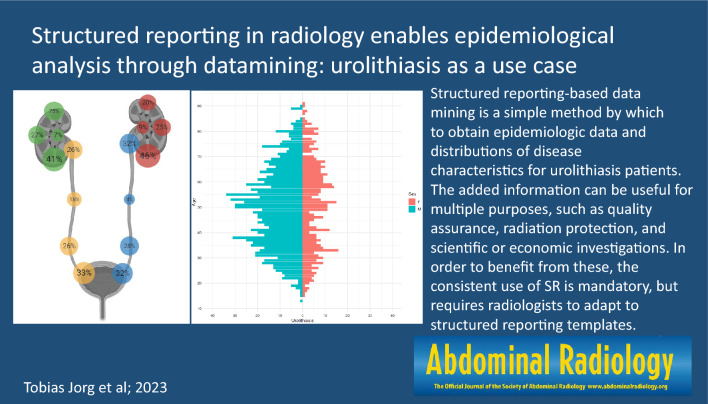

## Introduction

Urolithiasis is a common urologic disorder with rising prevalence over the last decades [1, 2]. It is estimated that in industrialized countries nearly 10% of the population is affected [3]. Although rarely life threatening it can cause intense pain and negatively affect patients’ quality of life. Hospitalization and recurrence rates are high [4]. Feared complications are renal failure and urinoma due to forniceal rupture [5]. Treatment options vary from medical expulsion therapy to operative placement of double-J catheters or percutaneous nephrolitholapaxy [4, 6, 7]. Due to its excellent sensitivity of over 90% in the detection of calculi, low-dose abdominopelvic CT is the method of choice for investigating patients with suspected urolithiasis [6, 7]. As number, size, location, and density of renal and ureteral calculi determine the ideal method of treatment for each individual patient, exact and correct radiological reporting of urolithiasis CTs is essential.

Most of these radiology reports are written as free text and lack structure [8, 9]. Furthermore, free-text reports are highly variable and often do not contain all the relevant information needed by the referring physician [10, 11]. Structured reporting (SR) in radiology is an emerging field of interest. In recent years, dozens of studies have been conducted to prove its benefits over classic free-text reporting (FTR). Among them are higher completeness and better report comparability and readability [9–12]. SR is the preferred form of radiology reporting for most referring physicians, including urologists [13]. Nevertheless, routine clinical SR usage is still low in most radiology departments. This might be attributed to the drawbacks of SR: the reporting of complex cases can be elaborate in template-based structured reports, and SR still lacks sufficient integration of speech recognition, which is commonly used for FTR [14]. Filling in SR templates using a mouse and keyboard can be time consuming and carries the risk of distraction from the image study [14, 15].

In addition to its advantages regarding the quality of the radiology report itself, SR as an IT-based method makes it possible to automatically acquire large structured datasets. These have enormous potential in data mining and epidemiological research [16–18]. The first steps in this field were done as a proof-of-concept study for pulmonary embolisms. At the time, structured reports were manually created from existing free-text reports, since SR had not yet been implemented in clinical routine at the corresponding institution [19]. Epidemiologic data derived from SRs that were done in real clinical routine are sparse.

Available imaging-based studies on the epidemiology of urolithiasis mostly deal with the distribution and size of ureteral calculi in smaller cohorts and were acquired by manually reviewing imaging datasets [20–22]. The existing larger epidemiologic studies, on the other hand, focus on patient characteristics and do not include CT imaging data [1, 3]. Overall, there is little clinical and healthcare research in the field of urolithiasis in comparison with cardiovascular diseases or cancer [4]. Because its high and rising prevalence causes a significant disease burden, including predicted costs of 4.5 billion dollar annually in the U.S. by 2030, further investigations are needed [23].

Thus, this study investigated the epidemiology as well as the distribution and characteristics of renal and ureteral calculi in urolithiasis by data mining the structured radiology reports of over 2000 patients who underwent abdominopelvic CT for urolithiasis.

## Methods

**Fig. 1:**
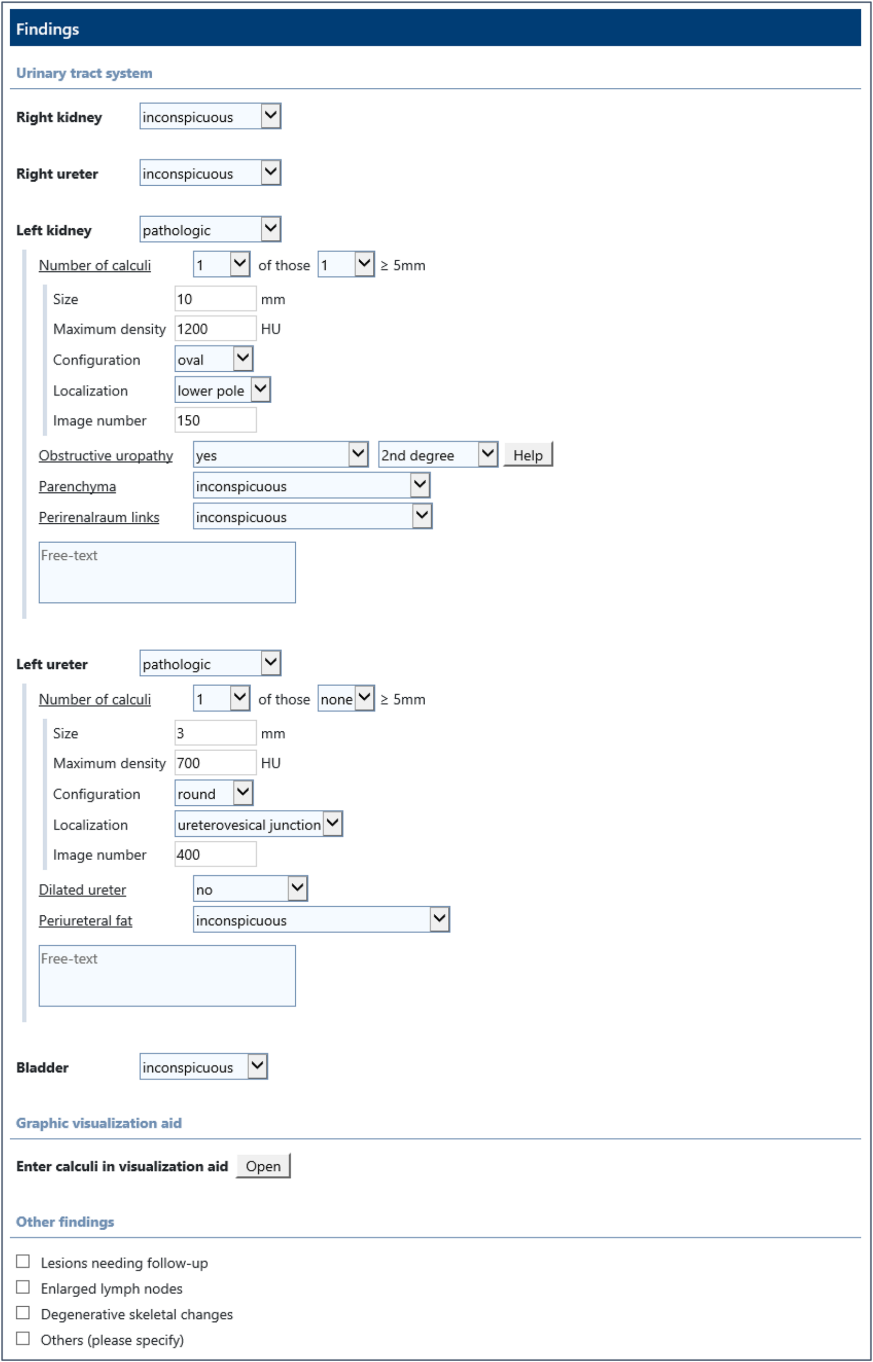
Screenshot of the SR template for urolithiasis CT (translated from German to English language). In this case a left-sided combined nephro- and ureterolithiasis was reported

At our department, an IHE MRRT-compliant web-based SR platform was developed and implemented into clinical routine in 2016 [24]. An SR template for urolithiasis CT was first introduced into clinical routine in September 2018. After a period of nearly 2 years, the template was further optimized in cooperation with the physicians from the department of urology and revised, with the new version introduced in May 2020. Fig. 1 shows a screenshot of the reporting template. In the reporting process, the templates are filled in using a mouse and keyboard. Measurements of calculus characteristics are made in the PACS and added to the report. A region of interest is manually set to determine the maximum density in Hounsfield Units (HU). For size measurement, the largest diameter is determined in MPR mode. In order to achieve a high degree of accuracy, instructions with measurement examples were provided for the reporting radiologists. For each organ system (left and right kidney, left and right ureter) the total number of calculi, including the number of those equal or larger than 5 mm, was documented. The calculus characteristics (maximum density, explicit size, location, and configuration) was only reported for the largest calculus in each organ system. In general, the reporting was done as an iterative process, in which the report was primarily created by a resident and then validated by a board-certified radiologist.

All CTs were performed with single energy scanners (Philips iCT, Amsterdam, the Netherlands). The scan protocol was always a low-dose, non-contrast abdominopelvic CT. The SR template was not applied to any other scan protocol.

We queried our SR database for all structured reports on urolithiasis CT done with the new version of the template from May 2020 to March 2023. The investigated patient cohort represents the full spectrum of a tertiary care center. It included patients presenting to the urology outpatient department with symptoms typical of urolithiasis (e.g., loin pain, colic, or hematuria) as well as symptomatic patients who presented primarily to the surgical or internal medicine emergency departments. Patients who were hospitalized at the time of the examination were also included.The content of all these reports, including the number, size, density, configuration, and location of calculi and possible obstructive uropathy, grade of uropathy, and organ characteristics for kidneys and ureters was extracted from the database using the software RapidMiner Studio 7.3 (RapidMiner, Cambridge, MA, USA). Patient age and sex were also extracted. All results were exported as a.csv file. Statistical analysis was performed using R version 4.2.2 (The R Foundation for Statistical Computing, Vienna, Austria). The prevalence of urolithiasis in general, defined as the proportion of reports that documented at least one renal or ureteral calculus, was calculated. Accordingly, the prevalences of nephrolithiasis, ureterolithiasis, and combined nephro- and ureterolithiasis were calculated. The sex and age distributions of urolithiasis were calculated. The distributions of calculus characteristics (size in mm, maximum density in HU, and number) were calculated. Furthermore, the distribution of calculi for each organ system (right and left kidney, right and left ureter) was determined. For patients with ureterolithiasis, the impact of calculus characteristics (location, configuration, density, and size) on the degree of possible obstructive uropathy was calculated. Results were reported either as absolute numbers and percentages or as mean values, standard deviation, median and interquartile range (IQR), and range. The mean completeness of the reports was calculated by subtracting the fields left blank from the total number of fields of the structured reports. Quality control was carried out by a board-certified radiologist who validated 100 randomly chosen reports for correctness by comparing them with the original image study.

## Results

A total of 2082 structured radiology reports of patients who underwent CT for urolithiasis were included in the analysis. 98 patients were examined more than once. In order to avoid over-representation only the reports of the chronologically first examination were included in the analysis. Of the 100 manually reviewed reports one had to be excluded due to minor quality.

Mean completeness of the included structured reports was 95 % (18.983 of 20.032 possible report fields were filled).

1883 (91 %) of the performed CT scans were ordered by urologists, 129 (6 %) by doctors from internal medicine and 70 (3 %) by surgeons. 1893 (91 %) patients primarily presented as outpatients while 189 (9 %) of the patients were hospitalized at the time of the examination (inpatients).

**Fig. 2:**
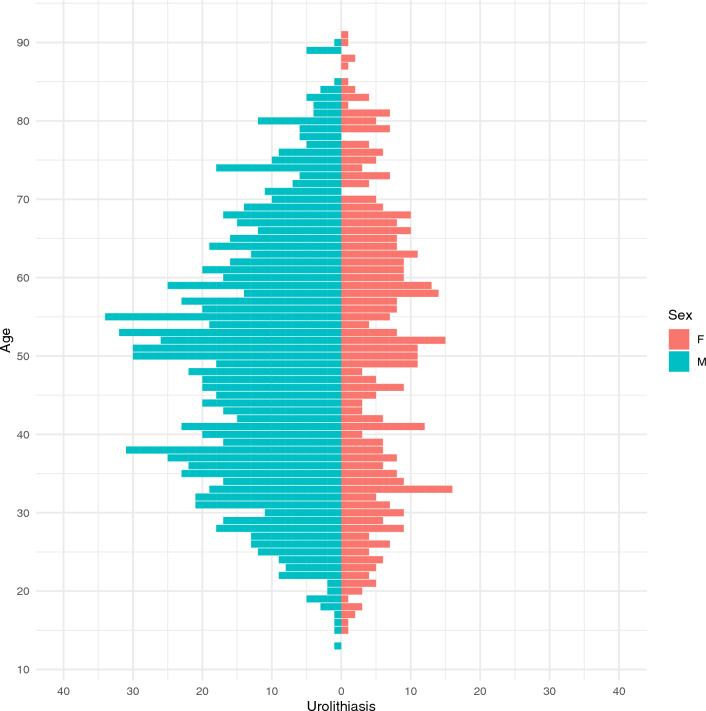
Sex and age distributions of urolithiasis

Of the 2082 examined patients 1504 (72 %) suffered from urolithiasis (defined as having at least one renal or ureteral calculus). Of those patients, 373 (25%) suffered from nephrolithiasis, 609 (40%) from ureterolithiasis, and 522 (35%) from combined nephro- and ureterolithiasis. The sex and age distributions of the patients are shown in Fig. 2. Urolithiasis was more frequently seen in men (M:F ratio, 2.3:1). The median patient age was 50 years (IQR 36–62).

**Fig. 3:**
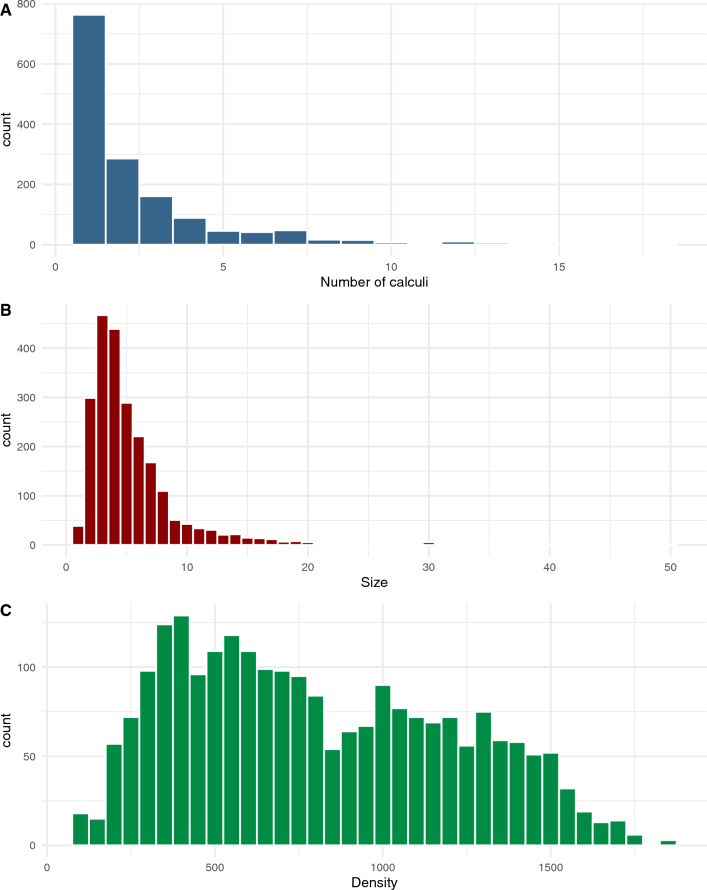
Distributions of the number of calculi (**A**), size of calculi (**B**), and maximum density of calculi (**C**)

The median number of calculi per patient was 1 (IQR 1–1, range 1–18, mean 2.5, SD 2.4) (Fig. 3A). The median calculus size, measured by the longest diameter in multi-planar reformation mode, was 4 mm (IQR 3–6, range 1–50, mean 5.2, SD 4.4) (Fig. 3B), and the median maximum density was 734 HU (IQR 465–1124, range 75–1864, mean 802.0, SD 403.0) (Fig. 3C).

**Fig. 4:**
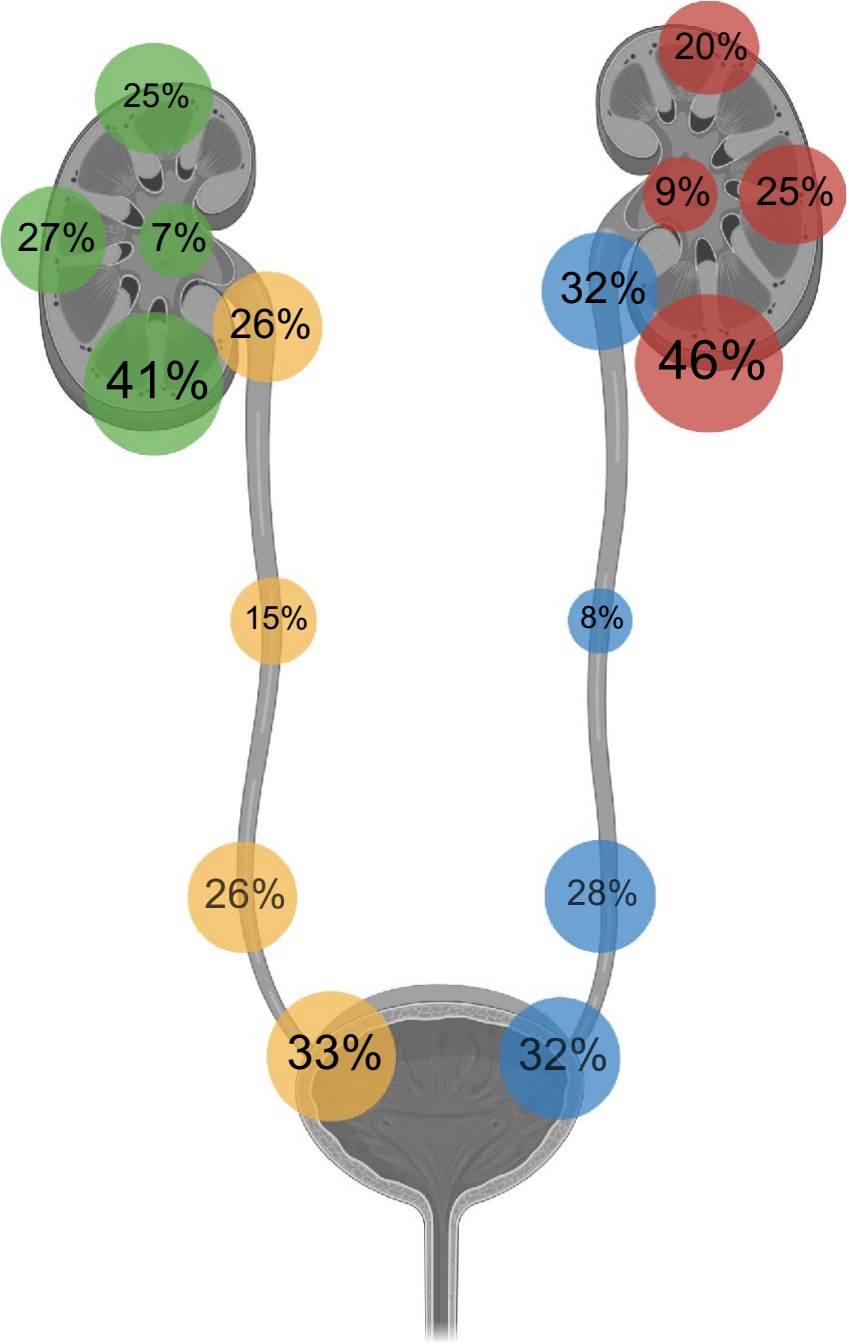
Distribution of calculi in each organ system. Right kidney (green), left kidney (red), right ureter (yellow) and left ureter (blue). The figure was created with Biorender.com

Regarding ureterolithiasis, the overall distribution of calculus location was as follows: 26% proximal, 15% mid, 26% distal, and 33% ureterovesical junction for the right ureter, and 32% proximal, 8% mid, 28% distal, and 32% ureterovesical junction for the left ureter. Renal calculi were distributed as follows: 25% upper pole, 27% interpolar, 41% lower pole and 7% renal pelvis for the right kidney, and 20% upper pole, 25% interpolar, 46% lower pole, and 9% renal pelvis for the left kidney. Fig. 4 shows the graphical distribution of calculi for each organ system.

**Fig. 5:**
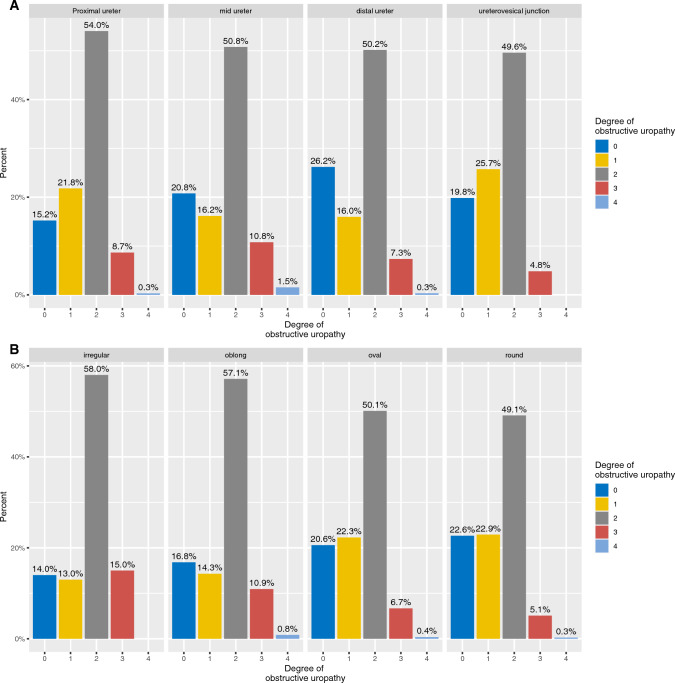
Impact of location (**A**) and configuration (**B**) of ureteral calculi on the level of obstructive uropathy

Of 1131 patients who had ureterolithiasis, 919 (81%) showed obstructive uropathy. Only 22 patients (2%) had bilateral ureteral calculi, while bilateral involvement was seen in 32% of patients with nephrolithiasis (287 of 895). For ureterolithiasis, the impact of the calculus location (proximal ureter, mid ureter, distal ureter, ureterovesical junction) and the calculus configuration (round, oval, irregular, oblong) on the level of obstructive uropathy is shown in Fig. 5. For all locations and configurations, 2nd-degree obstructive uropathy was the most common.

A urinoma was suspected in 36 patients (2% of all urolithiasis patients), while unspecific perirenal fat strandings were described in 527 patients (35%).

## Discussion

The results show that SR-based data mining for the creation of epidemiologic studies is feasible for the investigated cohort of urolithiasis patients without much expenditure of time or resources, provided that a detailed SR template has been consistently used for reporting beforehand It adds a valuable overview of the patient population, including detailed information on the distribution and characteristics of renal and ureteral calculi, which is helpful for both radiologists and clinical referrers.

In the investigated cohort, the median age of urolithiasis patients was 50 (IQR 36–62) and the sex distribution was 2.3:1 (M:F). This coincides with a recent population-based study describing the male-to-female ratio as nearly 3 and the peak patient age as 50–69 years [1]. The prevalence of urolithiasis was 72% in the investigated cohort. This value is consistent with another CT-imaging–derived study done with a smaller cohort [8]. However, there may be a selection bias for the high prevalence, since most of the examined patients showed symptoms typical of urolithiasis. In ureterolithiasis, calculi were most commonly seen at the ureterovesical junction. This confirms the findings of several other studies that manually reviewed smaller numbers of urolithiasis CTs (*n* ≤ 246) [21, 22, 25]. Published data on the distribution of renal calculi are limited. In our study, nephrolithiasis was most common at the lower poles of the kidneys.

Previous studies on the data mining of structured radiology reports were performed by retrospectively generating structured data from existing free-text reports [19]. To our knowledge, this is not only the first study that provides epidemiologic disease data derived from structured reports that were acquired in real clinical life, but it also analyses the largest cohort (*n* = 2082) investigated so far.

Besides the SR-based approach, data mining for epidemiologic purposes from free-text radiology reports is also possible through the use of natural language processing (NLP). NLP, as an artificial intelligence (AI)-related technology, is able to automatically structure and analyse free text [8]. A recent study showed that OpenAI’s NLP-based chatbot GPT-4 can be leveraged to retrospectively transform whole free-text radiology reports into structured reports [26]. Compared with the NLP-based approach, an SR-based approach for data mining radiology reports is much simpler and more time efficient. First, although NLP tools have improved in recent years, complete detection of all relevant information from free text is not guaranteed [27]. Second, the analysed free-text reports often lack relevant information, which further hinders the creation of complete datasets [10, 11]. In contrast, SR offers more complete reports that are primarily delivered in a highly structured form, making evaluations easier.

For SR-based data mining it is crucial that the level of SR usage in clinical routine is high, since reports that are still done in free-text form are not included in the analysis. Even though SR usage is not mandatory at our institution, we could demonstrate high use ratios for most examinations. This included urolithiasis CT, for which 91% of all reports were done as structured reports in 2022 [13]. Nevertheless, since SR is not equally applicable to all kinds of examinations, there might be differences in the suitability of the SR-based data mining approach, depending on the examination type.

The high prevalence of urolithiasis shown here speaks to the excellence of the indication checks prior to CT examination by the referring physicians. Knowing the expected rate of positive findings for examinations using ionizing radiation is important for both radiologists and referring physicians. In case of low rates, process optimization measures, like the development of new predictive scores, would be necessary [28]. SR-based data mining is a simple method with which to acquire such data for any examination and can therefore contribute to radiation protection and quality assurance.

Urologists can further benefit from knowing the distribution of disease characteristics like the size, maximum density, and location of renal and ureteral calculi. Since these characteristics determine if a patient receives conservative or operative treatment, knowledge of how many patients are to be expected for each treatment modality over a defined period of time can facilitate economic planning.

Moreover, structured data derived from CT reports can be used to support national disease registries for urolithiasis like the *Registry for Stones of the Kidney and Ureter* (ReSKU) or the *Register für Reccurente Urolithiasis* (RECUR) which are currently being implemented in the U.S. and Germany [4, 29]. Adding imaging-derived values such as calculus characteristics to the detailed patient data of those registries would enhance their informative value and could further enable the development of AI models—for example, for the prediction of recurrence risks. The provided data is a first step towards the integration of structured imaging data into disease registry databanks.

This study has several limitations. First, epidemiological data derived from CT examinations only allow an analysis of the disease at a defined point in time, even though urolithiasis is a dynamic process. Conclusions about the course of the disease cannot be drawn. Additionally, since the cohort represents the full spectrum of a tertiary care center. It included mostly symptomatic outpatients as well as inpatients. No asymptomatic patients were included and no patients receiving other imaging than CT for diagnosis (e.g., ultrasound) were included. Both factors hamper the clinical significance of the obtained epidemiologic data.

Second, the data mining approach is only easily feasible if a large number of structured reports is available. However, the time and effort required by radiologists to create these in clinical routine must be taken into account. Since the templates have to be filled manually by using a mouse and keyboard, it is possible that this is more elaborate than the creation of standard free-text report.

Third, clinical data and lab results were not taken into account. We are currently working on an interface between our SR platform and the lab software that would enable automated integration of lab results such as C reactive protein, creatinine, and white blood cell count into the structured radiology report. Since inflammation markers help to identify high-risk urolithiasis patients who require prompt treatment, their integration into the report can be of great value to the urologist [30]. Moreover, exact data on the prevalence of elevated inflammation markers in urolithiasis patients is sparse and could, again, easily be calculated with our data mining approach, once they are integrated into the structured report.

Lastly, the present study looks at the catchment area of a federal state in central Europe. However, the epidemiology of urolithiasis shows country- and continent-specific differences that are not reflected here. It is therefore all the more important that SR usage is further disseminated and introduced in more radiology departments all over the world. Multicentre data in a highly structured form could easily be used to analyse the regional differences in epidemiology.

## Conclusion

SR-based data mining is a simple method with which to obtain important epidemiologic data and disease characteristics, for the investigated cohort of urolithiasis patients. This information can be helpful for multiple purposes such as quality assurance, radiation protection, and scientific and economic investigations. These possibilities add to the long list of advantages of SR over FTR and underline the necessity of SR usage. However, SR usage in actual clinical routine may be elaborate since it requires radiologists to adapt to reporting templates that have to be filled using a mouse and keyboard.

## Data Availability

The datasets used for this study are available from the corresponding author on reasonable request.

## Abbreviations

SR: Structured reporting
FTR: Free-text reporting
NLP: Natural language processing
AI: Artificial Intelligence
RECUR: Register für reccurente urolithiasis; Registry for recurrent urolithiasis
ReSKU: Registry for stones of the kidney and ureter
IQR: Interquartile range
